# Inconsistencies in the sensitivity and specificity values in an Review Paper published in the Journal of Clinical Hypertension

**DOI:** 10.1111/jch.14353

**Published:** 2021-08-21

**Authors:** Michael S. Ortiz

**Affiliations:** ^1^ St. Vincent's Clinical School School of Medicine University of NSW Darlinghurst, NSW Australia

To the Editor

A research Review Paper published in your Journal^1^ reported on the use of the 8‐item Morisky Medication Adherence Scale (MMAS‐8) as a diagnostic tool to identify patients with uncontrolled hypertension (BP > 140/90 mm Hg) who were treated with medication for hypertension. This Review Paper contains mathematically implausible results for the Sensitivity and Specificity (S&S) based on the following evidence:

1. The publication described a “**sensitivity of 93%** ^1^” with a specificity of **53%** (pubS&S) and an accuracy of **80**% that “indicates that the scale is good at identifying patients who have low medication adherence and have uncontrolled blood pressure”.^1^
2. Using the 3 × 2 percentage data in Table 1 to calculate number of people in each medication adherence category (page 4 of the text) and blood pressure control (Table 4 on page 13), it is possible to reconstruct the study patient numbers by category. These values: 295, 144; 486, 482, are shown in the 2 × 2 table of Table 1. These data were used to calculate the S&S (calcS&S) values of **38%** [95%CI: 34–41%] and **75%** [72–79%], respectively, with an accuracy of **53%** [51.3–56.5%].
3. These values are remarkably similar to those published in a 2017 systematic review of the accuracy of the MMAS‐8^2^ (pooled S&S of: **43%** [33–53%] and **73%** [68–78%], respectively).
4. Based on the sensitivity, specificity, and accuracy, it is possible to derive a set of numerical values used to calculate the pubS&S. The 2 × 2 table of the *unique* mathematical solution includes: **858, 209; 65, 236**. These numbers were independently verified against the pubS&S values of 93% and 53%, along with accuracy of 80%.

**TABLE 1:** Patient numbers used to calculate the calcS&S values

(3 × 2) percentage data from the study				(2 × 2) reconstructed study population		
Test: Adherence	BP controlled			Test: Adherence	BP controlled	
MMAS‐8 score	No^a^	Yes	% total^b^	MMAS‐8 score	No	Yes
Low (< 6)	**67.2%**	**32.8%**	**32.1%**	Low (< 6)	**295**	**144**
Medium (6– < 8)	**55.2%**	**44.8%**	**52.0%**	Adherent (6–8)	**486**	**442**
High (8)	**43.3%**	**56.7%**	**15.9%**			
chi‐square	37.4	*p* < .001	*n* = 1367	chi‐square	26.2	*p* < .001

*Source*: Patient numbers were derived by multiplying proportions in the Review Paper by the number of patients and pooling by groups as shown.

^a^ From Table 4 page 13.

^b^ Text on page 4.

The pubS&S values (Point 1) differed markedly from the calcS&S values (Point 2). All the calculations were verified as being correct; however, the pubS&S number of non‐adherent patients (score < 6) is mathematically implausible. *This indicates that the pubS&S values are not consistent with the patient level data*.

1. The chi‐square value of 6.6 (*p* < .05) in Table 4^1^ is an obvious error as it does not reflect the 37.4 (p < 0.001) from the 3 × 2 data in Table 1. The study chi‐square value of the 2 × 2 patient numbers in Table 1 is 26.2 (*p* < .001), while the chi‐square value of the 2 × 2 patient numbers from the pubS&S (point 4) is much higher at 367.4 (*p* < .001).

So, not only is the chi‐square value in Table 4 incorrect, but also the difference between the latter two chi‐square values indicates that the pubS&S clearly overstates, by an order of magnitude, the diagnostic properties of the MMAS‐8 compared with the calcS&S values.

This study has a high impact in the Medication Compliance literature (cited > 3000 times in Pubmed). It is important for readers to be confident in the integrity of this Review Paper and that the published results are independently verified using the real data.

In conclusion, the S&S and accuracy values reported in this paper^1^ are mathematically implausible. The pubS&S values are inconsistent with the patient data and they overstate the diagnostic properties of the MMAS‐8. Unless these inconsistencies are resolved, it would appear that, based on the study results in Point 2 and the meta‐analysis,^2^ the MMAS‐8 scores may be no more accurate in detecting patients with uncontrolled BP, than tossing a coin to decide.

## CONFLICT OF INTEREST

The author has no competing interests.

## References

[1] Morisky DE , Ang A , Krousel‐Wood M , Ward HJ . Predictive validity of a medication adherence measure in an outpatient setting. J Clin Hypertens (Greenwich). 2008;10(5):348‐354. 18453793 10.1111/j.1751-7176.2008.07572.x PMC2562622

[2] Moon SJ , Lee WY , Hong YP , Morisky DE . Accuracy of a screening tool for medication adherence: a systematic review and meta‐analysis of the Morisky medication adherence Scale‐8. PLoS One. 2017;12(11):e0187139. 29095870 10.1371/journal.pone.0187139 PMC5667769

